# Transcriptomics and translatomics identify a robust inflammatory gene signature in brain endothelial cells after ischemic stroke

**DOI:** 10.1186/s12974-023-02888-6

**Published:** 2023-09-11

**Authors:** Maria Arbaizar-Rovirosa, Mattia Gallizioli, Juan J. Lozano, Julia Sidorova, Jordi Pedragosa, Sara Figuerola, Nerea Chaparro-Cabanillas, Patricia Boya, Mariona Graupera, Marc Claret, Xabier Urra, Anna M. Planas

**Affiliations:** 1 Department of Neuroscience and Experimental Therapeutics, Instituto de Investigaciones Biomédicas de Barcelona (IIBB), Consejo Superior de Investigaciones Científicas (CSIC), Rosselló 161, Planta 6, 08036 Barcelona, Spain; 2 Cerebrovascular Research Group, Institut d’Investigacions Biomèdiques August Pi I Sunyer (IDIBAPS), Barcelona, Spain; 3 University of Barcelona, Barcelona, Spain; 4 Bioinformatics Platform, Centro de Investigación Biomédica en Red Enfermedades Hepáticas Y Digestivas (CIBEREHD), Barcelona, Spain; 5 Department of Neuroscience and Movement Science, University of Friburg, Fribourg, Switzerland; 6 Endothelial Pathobiology and Microenvironment, Josep Carreras Leukaemia Research Institute, Barcelona, Spain; 7 Centro de Investigación Biomédica en Red de Cáncer (CIBERONC), Instituto de Salud Carlos III, Madrid, Spain; 8 Neuronal Control of Metabolism (NeuCoMe) Laboratory, Institut d’Investigacions Biomèdiques August Pi I Sunyer (IDIBAPS), Barcelona, Spain; 9 Centro de Investigación Biomédica en Red de Diabetes y Enfermedades Metabólicas Asociadas (CIBERDEM), Barcelona, Spain; 10 Unitat Funcional de Patología Vascular Cerebral, Hospital Clínic, Barcelona, Spain

**Keywords:** Ischemic stroke, Microvasculature, RiboTag, Fluorescence-activated cell sorting, Inflammation, Lipids

## Abstract

Vascular endothelial function is challenged during cerebral ischemia and reperfusion. The endothelial responses are involved in inflammatory leukocyte attraction, adhesion and infiltration, blood–brain barrier leakage, and angiogenesis. This study investigated gene expression changes in brain endothelial cells after acute ischemic stroke using transcriptomics and translatomics. We isolated brain endothelial mRNA by: (i) translating ribosome affinity purification, enabling immunoprecipitation of brain endothelial ribosome-attached mRNA for translatome sequencing and (ii) isolating CD31^+^ endothelial cells by fluorescence-activating cell sorting for classical transcriptomic analysis. Both techniques revealed similar pathways regulated by ischemia but they showed specific differences in some transcripts derived from non-endothelial cells. We defined a gene set characterizing the endothelial response to acute stroke (24h) by selecting the differentially expressed genes common to both techniques, thus corresponding with the translatome and minimizing non-endothelial mRNA contamination. Enriched pathways were related to inflammation and immunoregulation, angiogenesis, extracellular matrix, oxidative stress, and lipid trafficking and storage. We validated, by flow cytometry and immunofluorescence, the protein expression of several genes encoding cell surface proteins. The inflammatory response was associated with the endothelial upregulation of genes related to lipid storage functions and we identified lipid droplet biogenesis in the endothelial cells after ischemia. The study reports a robust translatomic signature of brain endothelial cells after acute stroke and identifies enrichment in novel pathways involved in membrane signaling and lipid storage. Altogether these results highlight the endothelial contribution to the inflammatory response, and identify novel molecules that could be targets to improve vascular function after ischemic stroke.

**Supplementary Information:**

The online version contains supplementary material available at 10.1186/s12974-023-02888-6.

## Introduction

The vascular endothelium is on the front line of the complex interface between the blood and the brain parenchyma that conforms the blood–brain barrier (BBB). Cerebral ischemia impacts upon the function of the different cellular components of the BBB and neuroinflammation plays a critical role in this process [1]. Ischemic stroke challenges the integrity of the BBB, allowing the passage of fluid from blood to brain leading to vasogenic edema [2]. Endothelial cells are affected by hypoxia, suffer different alterations and degenerate under severe ischemic conditions [3]. The response to hypoxia upregulates pro-angiogenic molecules, such as vascular endothelial growth factor (VEGF), which also promotes BBB leakage in the acute phase of stroke [4]. Endothelial cells display changes in gene and protein expression after ischemia that contribute to endothelial dysfunction, mediate leukocyte attraction, adhesion, and infiltration to the ischemic tissue, and participate in basal lamina remodeling and angiogenesis. The discovery of novel molecular changes induced by ischemia in endothelial cells may contribute to the development of more personalized treatments aimed at improving the current benefits of reperfusion therapies. Moreover, identification of new endothelial surface molecules upregulated by ischemia could enable functionalizing therapeutic nanoparticles with specific antibodies to guide them to the affected brain vessels.

Phenotypic alterations are often regulated at the transcriptional level. Recent single-cell transcriptomics provided relevant information regarding vessel heterogeneity and arterio-venous zonation in the brain [5]. A previous work studied the transcriptomic profile of brain endothelial cells in various disease models, including a model of permanent ischemia [6]. Interestingly, this study reported a BBB dysfunction gene module, common to various experimental disease situations, that caused a shift of the endothelial brain transcriptomic profile towards a pattern resembling a peripheral endothelial cell-like state [6]. A recent study evaluated the effect of transient ischemia on the transcriptomic profile of the cellular components of the neurovascular unit after obtaining vascular endothelial (VE)-cadherin^+^ cells by cell sorting [7]. These studies illustrate how transcriptomics may help to identify molecules that are relevant for vascular disease conditions. However, classical transcriptomic studies face several technical problems. First, global mRNA is not a faithful representation of the proteins that are synthesized, whereas they are more closely represented by the mRNA associated with ribosomes, which is the mRNA being translated that collectively conforms the translatome [8–10]. Second, the isolation of specific cell types from tissues requires obtaining single cell suspensions, which need long procedures and several treatment steps, such as enzymatic digestion, that may induce artifacts in the recovered mRNA transcripts [9]. Obtention of ribosome-associated mRNA using genetic tools, like a translating ribosome affinity purification method using the RiboTag mice [10], may partially circumvent these problems [8–10].

The current work was aimed to identify pathogenic responses and gene expression changes involved in endothelial dysfunction caused by cerebral ischemia/reperfusion. We isolated mRNA from brain endothelial cells through two different strategies: (i) the RiboTag method using platelet-derived growth factor *Pdgfb*^*icreER*^*:Rpl22*^*HA*^ mice and (ii) cell sorting of CD31^+^ endothelial cells. We defined a robust endothelial cell translatome module responsive to acute ischemic stroke compiling the differentially expressed genes (DEGs) that were common in both strategies. Co-expression network analysis enabled to define gene clusters associated with functional processes and hub genes that could play regulatory functions. Our study identified molecules that may open new avenues to understand and intervene on the endothelial responses to brain ischemia.

## Materials and methods

### Animals

We used C57BL/6 mice (3–4 months of age) maintained at the animal facility at the School of Medicine of the University of Barcelona under standardized conditions. We used male mice in the RNA study, and we used male and female mice in the protein validation studies. RiboTag mice B6N.129-Rpl22tm1.1Psam/J (# 011029, Jackson Laboratories, previously crossed to the C57BL/6 background for 9 generations), which carry a conditional Rpl22 allele [10], were crossed with *Pdgfb*^*iCreER*^ mice (C57BL/6 background) harboring a tamoxifen-inducible form of Cre recombinase in vascular endothelial cells [11] (kindly provided by Dr. Marcus Fruttiger), to generate *Pdgfb*^*icreER*^*:Rpl22*^*HA*^ mice.

### Brain ischemia

To induce cerebral ischemia/reperfusion, mice were anesthetized with isoflurane and received analgesia (buprenorphine, 140 µl of a 0.015 mg/mL solution, i.p.). We occluded the middle cerebral artery (MCA) with an intraluminal filament (#701912PK5Re, Doccol Corporation) and monitored the blood flow drop as described [12]. At 45 min of the MCA occlusion, the filament was removed, the wound was sutured, anesthesia was withdrawn, and mice were kept on a thermal blanket at 37 °C for 1 h to facilitate recovery. Sham-operated mice were subjected to all surgical and anesthetic procedures but the filament was not inserted in the MCA. Mice were anesthetized with isoflurane and received an MRI scan to image the brain lesion 1-day post-ischemia in a 7.0 T BioSpec 70/30 horizontal animal scanner equipped with a 12-cm inner diameter actively shielded gradient system (400 mT/m). The receiver coil was a phased array surface coil for mouse brain (Bruker BioSpin, Ettlingen, Germany). We used a T2w turbo RARE fast spin-echo MRI sequence with 1 effective echo time (ET) = 33 ms, slice thickness = 0.5 mm, repetition time (TR) = 2336 ms, field of view = 20 × 20 mm^2^, matrix size = 256 pixels, and in-plane spatial resolution = 0.078 mm. After MRI, the mice were euthanized under deep isoflurane anesthesia.

### Tamoxifen treatment of PdgfbicreER:Rpl22^HA^ mice

Mice received intraperitoneal injections of 100 µl of Tamoxifen (#T5648, Sigma Aldrich) at a concentration of 20 mg/ml diluted in Corn Oil (#C8267, Sigma Aldrich) for 5 consecutive days, reaching a total amount of 10 mg/mouse.

### RiboTag strategy to obtain mRNA attached to ribosomes of brain endothelial cells

The RiboTag method [10] is used to immunoprecipitate ribosomes from endothelial cells using transgenic *Pdgfb*^*icreER*^*:Rpl22*^*HA*^ mice. In this model, ribosomal subunit *Rpl22* is flanked by two *loxP* sites enabling hemagglutinin (HA)-tagged Rpl22 expression in cells expressing *Pdgfb*^icreER^ after tamoxifen administration. Tagged-ribosome immunoprecipitation using anti-HA antibodies allows obtaining the ribosome-attached mRNA. Brain tissue samples were dissected in different regions, i.e., cortex and striatum from both the ipsilateral (ischemic) and contralateral hemispheres (control). The tissue was homogenized in 3% (weight–volume) buffer containing 1.5 M TRIS pH 7.4, 1 M KCl, 1 M MgCl_2_ and 10% Igepal (#CA-630, Alfa Aesar) supplemented with 1M 1,4-Dithiothreitol (#3483-12-3, DTT Sigma Aldrich), protease inhibitors (#P8340, Sigma), 200 UI/ml RNAsin (#N2115, Promega), 5 mg/mL cycloheximide (#C1988, Sigma) and heparin (ROVI, 5000 Ul/mL). Homogenization was performed in a 7 mL-glass Douncer homogenizer (#9063, Sigma) passing the pestle-A 15 times and 15 times more with the pestle-B. Then, the lysate was incubated in rotation at 4 °C with 2 mg/mL mouse monoclonal (IgG1, κ) anti-HA antibody (HA.11 Epitope Tag, clone 16B12, #901513, Biolegend) for 3 h. Then, 300 μl Dynabeads Protein G (#10004D, Thermo Fisher Scientific) were added and the mix was incubated 2 more hours in the same conditions. Finally, a magnetic stand (#10723874, Invitrogen—DynaMag™-2) was used to remove all the non-bound sample. Three washes with a high-salt buffer were performed to ensure specific binding of the beads to our target sample. Lysis buffer (#74034, Qiagen) supplemented with 10% β-mercaptoethanol was added to elute the sample. Finally, the sample was separated from eluted magnetic beads by strong vortex for 30 s. Total RNA was purified with the RNeasy Plus Micro Kit (#74034, Qiagen) for elimination of genomic DNA. RNA was precipitated with 80% ethanol and the RNA was eluted with H_2_O RNase-free. We focused our RNA analyses on the cortex for technical reasons related to obtention of higher RNA content from cortex than striatum due to the larger size of the former region.

### Immunofluorescence in brain tissue sections

Mice were perfused through the heart with 20 mL of cold heparinized phosphate-buffered saline (PBS) followed by 20 mL of cold 4% paraformaldehyde diluted in phosphate buffer (PB) pH 7.4. The brain was removed, fixed overnight with the same fixative, and immersed in 30% sucrose in PB for cryoprotection for at least 48 h until the brains were completely sunk to the bottom of the tube. Brains were frozen in isopentane at – 40 °C. Cryostat brain section (14-μm thick) were fixed in ethanol 70%, blocked with 3% donkey serum, and incubated overnight at 4ºC with mouse monoclonal (IgG1, κ) anti-hemagglutinin (HA.11 Epitope Tag) antibody (clone 16B12, #901513; RRID:AB_2565335) diluted 1:500, followed by Alexa Fluor™ Plus 488 secondary antibody (#A32766, Invitrogen, RRID:AB_2762823) diluted 1:500. We labelled the blood vessels using biotinylated Lycopersicon esculentum lectin (#NC9862633, Fisher Scientific) diluted 1:400 followed by Streptavidin, Alexa Fluor™ 546 conjugate (#S11225, ThermoFisher) diluted 1:500. Double immunofluorescence was carried out for HA and markers of microglia (Iba-1, 1:400, #019-19741, RRID:AB_839504, Fujifilm Wako Pure Chemicals Corp.) or neurons (NeuN, 1:400, #ab177487, RRID:AB_2532109, Abcam). Cell nuclei were stained with DAPI (Invitrogen). Images were obtained in a confocal microscope (Dragonfly, Andor).

To assess BBB integrity, we obtained vibratome brain section (30-μm thick) that were permeabilized with 0.2% triton x-100 in PBS, blocked with 3% donkey serum and incubated free-floating overnight at 4 °C with Alexa Fluor 488 Donkey anti-Mouse IgG (1:500, #A32766, Life Technologies S.A., RRID:AB_2762823) and a polyclonal antibody against Glut1 (1:500, #07-1401, Merck-Millipore, RRID:AB_1587074). The secondary antibody was Alexa Fluor 555 Donkey anti-rabbit (1:500, #A32794, Life Technologies S.A., RRID:AB_2762834). Images of the entire sections (*n* = 3 per mouse) were obtained with the 4 × objective of a microscope (Olympus BX51) with motorized stage (Prior Pro Scan II) and equipped with a digital camera (Olympus DP71).

Some of the new genes identified by RNAseq were validated at the protein level by immunofluorescence in vibratome brain sections incubated as above with a rat monoclonal antibody anti-CD146 (1:50, clone ME-9F1, #134713, Biolegend, RRID:AB_2563108) followed by a secondary antibody Alexa Fluor® 594 (1:500, #ab150156, Abcam, RRID:AB_2890252), and a rat monoclonal anti-CD155 antibody (1:40, clone TX56, BV421, #131517, Biolegend, RRID:AB_2716159) followed by Alexa Fluor™ Plus 488 (1:500, #A48269, Invitrogen, RRID:AB_2893137) secondary antibody. These immunoreactions were combined with a rabbit polyclonal anti-Glut-1 antibody, as above (1:500, RRID:AB_1587074), to visualize the blood vessels. Finally, we used a rabbit polyclonal anti-Plin2 antibody (ADPF, 1:200; # PA1-16972, RRID:AB_2223607, Invitrogen) followed by Alexa Fluor™ Plus 488 (1:500, #A32790, Invitrogen, RRID:AB_2762833) secondary antibody. In this case, blood vessel location was seen with a goat polyclonal antibody against α4-laminin (1:100, #AF3837, R&D Systems, RRID:AB_2249744) followed by a secondary antibody Alexa Fluor™ 546 (#A11056, Invitrogen, RRID:AB_2534103) diluted 1:500. Images were obtained in a confocal microscope (Dragonfly).

Image analysis of CD146 and CD155 staining was performed with Fiji software. Quantification of mean fluorescence intensity (MFI) was carried out by obtaining three images (X63 magnification: 210 × 209 μm^2^) in a brain section of sham-operated mice, and ischemic mice at days 1 and 4 post-ischemia. In brief, we generated a z-projection for each fluorescence channel. Segmentation was performed in the channel of stained blood vessels and the corresponding mask was applied to the CD146 or CD155 channel to measure the MFI.

### Oil Red O staining 0.3%

Oil Red O (0.5%, #O1391-250ml, Sigma‐Aldrich) was diluted to 0.3% working solution in isopropanol stock solution in a ratio 3:5 with milliQ water. The solution was let stand for 10 min at room temperature and then filtered through a 22 μm filter. Vibratome brain section (30 μm-thick) were washed 3 times in PBS for 5 min, followed by a 2 min incubation in 60% isopropanol and then stained with freshly prepared Oil Red O working solution for 15 min at room temperature. Sections were rinsed 3 times for 5 min with deionized water and mounted with an aqueous medium (#F4680-25ml, Sigma‐Aldrich). Finally, the images were captured using an Olympus BX51 microscope.

### Brain tissue processing for cell sorting and flow cytometry

The brain tissue was processed as previously reported with modifications [13]. The brain was carefully collected and immersed in Hanks’ Balanced Salt solution w/o ions (HBSS w/o Ca^2+^ and Mg^2+^; #14175-053, Thermo Fisher Scientific) on ice. The forebrain was dissected, discarding the cerebellum and the olfactory bulbs, and the ipsilateral brain hemisphere was minced in small pieces with a scalpel. The Neural Tissue Dissociation Kit—P (NTDK, #130-092-628, Miltenyi Biotec) was used to homogenize the tissue in C-tubes (#130-093-237, Miltenyi Biotec) with mechanical dissociation by gentleMACS™ Octo Dissociator (#130-096-427, Miltenyi Biotec: 1 × m_Brain_1 program and 1 × ABDK_37C program by Miltenyi), according to manufacturer instructions. The tissue was then filtered through a 70 μm cell strainer (#352350, Falcon) and washed with HBSS with Ca^2+^ and Mg^2+^ (#14025-092, Thermo Fisher Scientific). Then, cells were separated from myelin by an immunomagnetic separation method. Brain cells were incubated with Myelin Removal Beads II (#130-096-733, Miltenyi Biotec) and then passed through LS Columns (#130-042-401, Miltenyi Biotec) held onto the OctoMACS Separator (#130-091-051, Miltenyi Biotec) and to the MACS® MultiStand (#130-042-303, Miltenyi Biotec), according to the manufacturer instructions. Unspecific binding of antibodies was blocked by previous incubation for 10 min with anti CD16/CD32 (Fc block, clone 2.4G2; #553142, BD Pharmingen, RRID:AB_394657) in Fluorescence Activated Cell Sorting (FACS) buffer at 4 °C. Live/dead staining (Live/Dead Fixable Blue Dead Cell Stain kit #L34961, Thermo Fisher Scientific) was used to determine the viability of cells. Cells were incubated during 30 min at 4 °C with the following primary antibodies: CD31 (clone 390, PE-Cyanine7, #25-0311-82, eBioscience, RRID:AB_2716949; or clone 390, BV421, #102423, Biolegend, RRID:AB_2562186), CD11b (clone M1/70, PE, #553311, BD Pharmingen, RRID:AB_394775), CD45 (clone 30-F11, FITC, #553080, BD Horizon, RRID:AB_394610), CD155 (clone TX56, BV421, #131517, Biolegend, RRID:AB_2716159), CD354 (clone 174031, BV711, #747902, BD Pharmingen, RRID:AB_2872364), CD146 (clone ME-9F1, PE-Cyanine7, #134713, Biolegend, RRID:AB_2563108), CD262 (clone MD5-1-3C2, PerCP-Vio700, #130-105-697, Miltenyi Biotec, RRID:AB_2656753). After a wash with cold FACS buffer, the cells were analyzed in a BD Fortessa 5L cytometer using FacsDiva software (version 5, BD Biosciences, San Jose, CA, USA). Data analyses were performed with FlowJo software (version 10, FlowJo LLC, Ashland, OR, USA). Gates were established using FMO as controls.

### Fluorescence activated cell sorting (FACS)

We obtained CD31^+^ endothelial cells by cell sorting, essentially as previously reported [14]. Unspecific binding was blocked by incubation for 10 min with anti CD16/CD32 (Fc block, clone 2.4G2; #553142, BD Pharmingen, RRID:AB_394657) in FACS buffer at 4 °C. Live/dead Aqua Dead Cell stain kit (#L34957, Thermo Fisher Scientific) was used to determine the viability of cells. Cells were incubated with the following primary antibodies during 30 min at 4 °C: CD11b (clone M1/70, APC-Cy7, #557657, BD Pharmingen, RRID:AB_396772), CD45 (clone 30-F11, FITC, #553080, BD Pharmingen, RRID:AB_394610), CD31 (clone 390, PE-Cyanine7, #25-0311-82, eBioscience, RRID:AB_2716949). After washing with FACS Stain Buffer (#554656, BD Biosciences), the cells were sorted in a FACSAriaII or FACSAria SORP sorter (BD Biosciences). Endothelial cells were collected in sterile DPBS (#14190-094, Thermo Fisher Scientific), centrifuged, and resuspended in lysis buffer (from PureLink™ RNA Micro Kit #12183016, Invitrogen) supplemented with 10% β-mercaptoethanol and finally snap-frozen in dry ice.

### RNA extraction from sorted cells

RNA was extracted from FACS endothelial cells with PicoPure™ RNA Isolation Kit (#KIT0204, Thermo Fisher Scientific). RNA was precipitated with 70% ethanol. To avoid genomic DNA contamination a DNAse step was performed using Invitrogen™ PureLink™ DNase Set (#12185010, Invitrogen). RNA quantity and purity were assessed with the Pico Kit Assay on the Agilent 2100 Bioanalyzer System.

### RT-PCR

We extracted RNA from whole brain tissue (cortex) using Trizol® Reagent (Life Technologies) followed by PureLink™ RNA Mini Kit (#12183018 A, Invitrogen). RNA quantity and quality were analyzed using ND-1000 micro-spectrophotometer (NanoDrop Technologies). One µg of total RNA was reverse transcribed using a mixture of random primers (#4387406, High-Capacity cDNA Reverse Transcription kit, Applied Biosystems), and the cDNA was diluted six times in RNAse-free water. The RNA obtained from FACS-sorted cells or immunoprecipitated-samples was analyzed with Qubit RNA Hs (Thermofisher), and 20ng or 2ng RNA, respectively, were reverse transcribed. The cDNA was pre-amplified using the TaqManVR Pre Amp Master Mix (2x) (#4384266, Applied Biosystems™) using a pool of TaqMan probes. The final product was diluted 20 times with tris–EDTA buffer pH 8.0 (#BP2473, Fisher Bioreagents). RT-PCR was carried out with Taqman™ system (#4440038, Applied Biosystems™) using iCycler iQ™ Multicolor Real-Time Detection System (Bio-Rad). We quantified by normalizing cycle threshold (Ct) values with Hprt1 housekeeping gene Ct. Fold enrichment analysis from endothelial samples was carried out with the 2 − ΔΔCT method referred to brain tissue samples. The Taqman probes used were as follows: Pecam1: Mm01242576_m1; Vegfc: Mm00437310_m1; Gfap: Mm01253033_m1; Pdgfrb: Mm00435546_m1; Flt1: Mm00438980_m1; Ackr1: Mm04207950_g1; Tmem119: Mm00525305_m1; Tubb3: Mm00727586_s1; Hprt1: Mm00446968_m1.

### RNA sequencing

cDNA was generated using Smart-seq2 protocol [15]. Briefly, total RNA was reverse transcribed using betaine and increasing the magnesium chloride concentration, template switching was performed using a locked nucleic acid and elimination of purification step before preamplification PCR to obtain an increased cDNA yield from 2 µl RNA. cDNA concentration was measured with Qubit dsDNA High Sensitivity assay (ref. Q32851, Invitrogen) and was analyzed using Agilent Bioanalyzer or Fragment analyzer High Sensitivity assay (ref. 5067-4626 or DNF-474, Agilent) to check size distribution profile. cDNA libraries were prepared using NEBNext® Ultra DNA Library Prep for Illumina® kit (ref. E7370) according to the manufacturer’s protocol. Briefly, 5 ng of cDNA were fragmented at a range size of 200–500bp using Covaris S2, they were subjected to end repair and addition of “A” bases to 3′ ends, ligation of adapters and USER excision. All purification steps were performed using AgenCourt AMPure XP beads (ref. A63882, Beckman Coulter). Library amplification was performed by PCR using NEBNext® Multiplex Oligos for Illumina (Index Primers Set 1, ref. E7335), (Index Primers Set 2, ref. E7500), (Index Primers Set 3, ref. E7710) or/and (Index Primers Set 4, ref. E7730). Final libraries were analyzed using Agilent Bioanalyzer or Fragment analyzer High Sensitivity assay to estimate the quantity and check size distribution, and were then quantified by qPCR using the KAPA Library Quantification Kit (ref. KK4835, KapaBiosystems) prior to amplification with Illumina’s cBot. Libraries were sequenced 1 × 50 + 8 bp on Illumina’s HiSeq2500.

### Transcriptomic analysis and statistics

For RNAseq data, processing of raw reads including quality control, trimming for Illumina adapters, filtering, alignment to mouse genome and gene quantification were performed as previously reported [12]. We used DESeq2 on raw counts for identifying differentially expressed genes [16]. Gene Ontology and Reactome canonical pathway enrichment analysis was performed through GSEA function in cluster Profiler package (gseGO and gsepathway for enrichment analysis and cnetplot graphics) [17] using computed Wald statistic. GOBubble function from GOPlot R package was used to represent the most enriched GO terms [18]. Heatmaps and Principal component plots were performed using R statistical software.

For other data, multiple groups were compared with Kruskal–Wallis test followed by Dunn’s multiple comparisons test. Paired two-group comparisons were carried out with the Wilcoxon matched-pairs signed rank test. We made estimations of sample size based on information on the group mean and SD from previous flow cytometry or RNA data of our own laboratory and we built from these data the number of animals needed for comparing groups with the minimum reasonable numbers of animals. The specific tests used in each experiment, p values, and n values are stated in the figure legends. We used GraphPad Prism software version 9.3.1.

## Results

### Translatomic profile of brain endothelial cells obtained with the Pdgfb^icreER^:Rpl22^HA^ mice

**Fig. 1:**
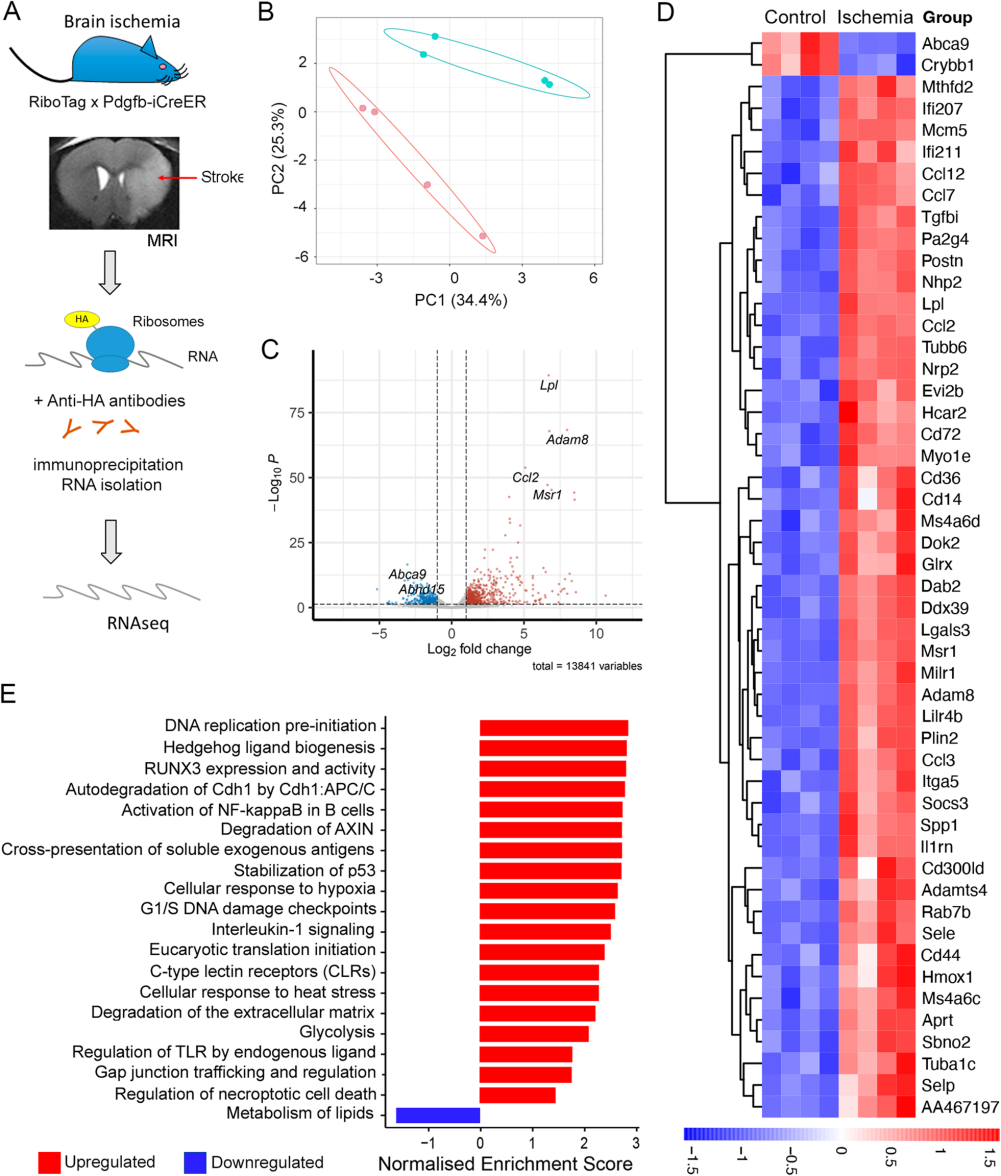
Ischemia-induced translatomic changes in mRNA obtained from the *Pdgfb*^*icreER*^*:Rpl22*^*HA*^ mice. **A** Mice were obtained by crossing *Pdgfb-iCreER mice* with the RiboTag mice. This strategy enables the obtention of ribosome-associated mRNA from Pdgfb^+^ cells through immunoprecipitation with anti-HA antibodies. Brain infarction after MCA occlusion was verified by MRI at 24 h. RNAseq from ischemic (ipsilateral) and corresponding control (contralateral) cortex 24 h post-ischemia. Image created with BioRender.com. **B** PCA shows clear separation of ischemic (green) from control (red) samples (*n* = 4 per group). **C** MA plot illustrating the log2-fold-change between ischemic and control groups against the average expression across all the samples for each gene, marking those genes with higher statistical significance using FDR < 0.05 and log2 fold change >|2|. Points show the genes upregulated (red) and downregulated (blue). N.S. indicates genes showing non-significant differences (grey). The top 5 genes leading in each direction are marked with their names. **D** Fifty top DEGs are shown. **E** Selection of pathways regulated by ischemia (full data sets in Additional file 2: Table S1)

We isolated RNA from brain endothelial cells by crossing the *Pdgfb*-icreER mouse [11] with the RiboTag mouse model [10] (Fig. 1A). This model enabled immunoprecipitation of the HA-tagged ribosomes and associated mRNA from cells expressing *Pdgfb*. Pdgfb is mainly expressed in capillaries and, to a lower extent, venules, and arterioles of the adult CNS [11]. We induced cerebral ischemia and confirmed by MRI that all mice showed brain infarction at 24h (Fig. 1A). The size of the MRI lesion was 55.8 ± 29.2 mm^3^ (mean ± SD, *n* = 4) (Additional file 1: Fig. S1A). We obtained RNA from the cortex and striatum through immunoprecipitation 24h post-ischemia and carried out RNAseq. Ischemic samples (ipsilateral) clearly separated from controls (contralateral) (Fig. 1B). The MA plot illustrates the most significant DEGs after ischemia (Fig. 1C) The top DEGs after ischemia were upregulated (Fig. 1D) and included mainly genes encoding inflammatory and innate immune molecules regulated by the type-I interferon (IFN) program. Global regulated genes after ischemia are shown in Additional file 1: Fig. S1B. Upregulated pathways highlighted inflammatory responses, antigen cross-presentation, DNA damage and repair, cell division, translation initiation, and cellular response to hypoxia, amongst others. One of the few pathways downregulated by ischemia was lipid metabolism (R-MMU-556833). Selected pathways are shown in Fig. 1E (see full list in Additional file 2: Table S1).

### Transcriptomic profile of brain CD31^+^ endothelial cells obtained by FACS

**Fig. 2:**
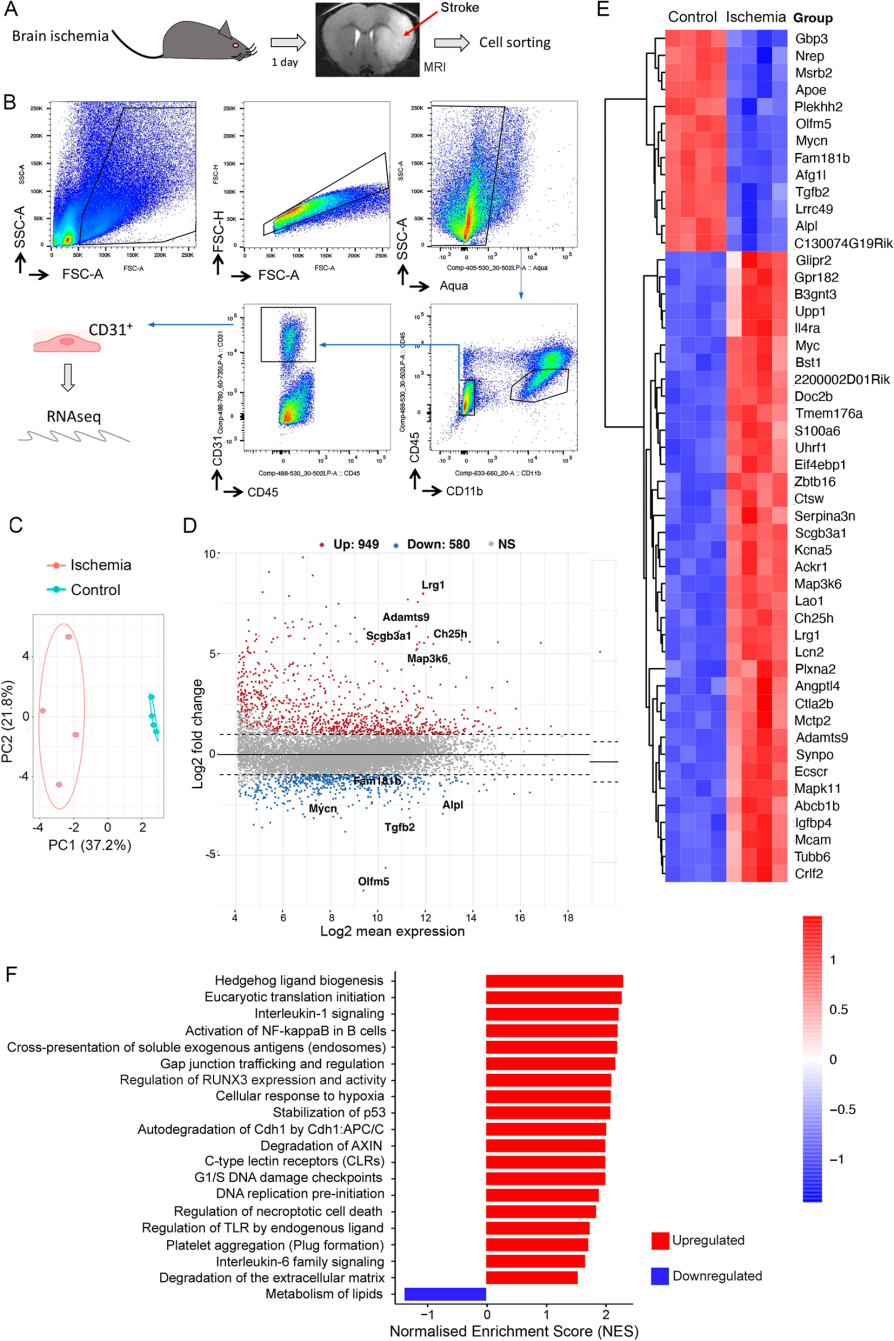
Ischemia-induced transcriptomic changes in CD31^+^ brain endothelial cells. **A** Brain infarction was verified by MRI 24h after MCA occlusion. Brain tissue was obtained from the right (ischemic) brain hemisphere at 24h, and from naïve mice. **B** FACS gating strategy used to isolate CD31^+^ endothelial cells, the RNA was extracted, and RNAseq was performed. **C** PCA shows clear separation of ischemic from control samples (*n* = 4 per group). **D** MA plot showing the genes with the highest differential expression and statistical significance using FDR < 0.05 and log2 fold change >|2|. Points show the genes upregulated (red) and downregulated (blue). N.S. indicates genes showing non-significant differences (grey). The genes that rank highest in each direction are tagged with their names. **E** Fifty top DEGs are shown, illustrating that they were mostly upregulated. **F** Selection of pathways regulated by ischemia (full data sets in Additional file 3: Table S2)

For comparative and validation purposes we obtained RNA from endothelial cells of wild type mice by means of a completely different experimental approach, based on isolation of CD31^+^ (*Pecam1*) endothelial cells by FACS from the cortex and striatum of naïve mice and 24h after transient brain ischemia (Fig. 2A, B). Brain infarction was assessed by MRI in all mice at 24h (Fig. 2A). The size of the brain lesion (mean ± SD, *n* = 4) was 41.9 ± 19.2 mm^3^ (Additional file 1: Fig. S1C). After isolating the CD31^+^ cells from the ischemic and control mice (Fig. 2b), we extracted the RNA and carried out a transcriptomic analysis by RNAseq. The PCA perfectly separated ischemic from control samples (Fig. 2C). The MA plot highlights the most significant DEGs after ischemia (Fig. 2D). The global heatmap of DEGs induced by ischemia in endothelial cells (Additional file 1: Fig. S1D), and the top 50 DEGs (Fig. 2E) illustrate upregulation of the majority of DEGs after ischemia. Pathway analysis (Fig. 2F, Additional file 3: Table S2) provided similar results as those obtained with the RiboTag technique (Fig. 1E, Additional file 2: Table S1), illustrating that main gene enrichment was consistent in both strategies.

### Enrichment of endothelial RNA in the two different preparations

In the RNAseq data sets obtained using the RiboTag or the CD31^+^ cell sorting approaches, we checked the expression of genes reported as endothelial cell restricted and brain tissue-specific [19]. We found most of the reported annotated genes in the mRNA from both techniques, but in general their expression was more enriched after cell sorting compared to the RiboTag preparation, where only the mRNAs being associated with the ribosomes were obtained (Additional file 4: Fig. S2A). Given the alteration of the BBB caused by brain ischemia (Additional file 4: Fig. S2B), we examined whether our data sets of DEGs in the ischemic endothelium contained genes previously described in a BBB dysfunction module [6]. This described gene module comprised 136 DEGs that were common to different pathological conditions, which shared the feature of inducing BBB breakdown [6]. After transient ischemia, we identified 74 genes upregulated in our cell sorting data set and 44 genes in our RiboTag data set common to this BBB-dysfunction module (Additional file 4: Fig. S2C–F). A study based on sorted vascular cadherin^+^ (*Cdh5*) cells recently reported DEGs in the endothelium 24h after transient ischemia [7]. We verified that the 15 top upregulated genes in the latter study [7] were also upregulated in our cell sorting study. However, only 7 of these genes (*Car13, Tubb6, Cd200, Adamts9, Adamts4, Amgpt2*, and *Hmox1*) were among the upregulated genes found in the RiboTag study, illustrating that the translatome is more restrictive than the transcriptome.

**Fig. 3:**
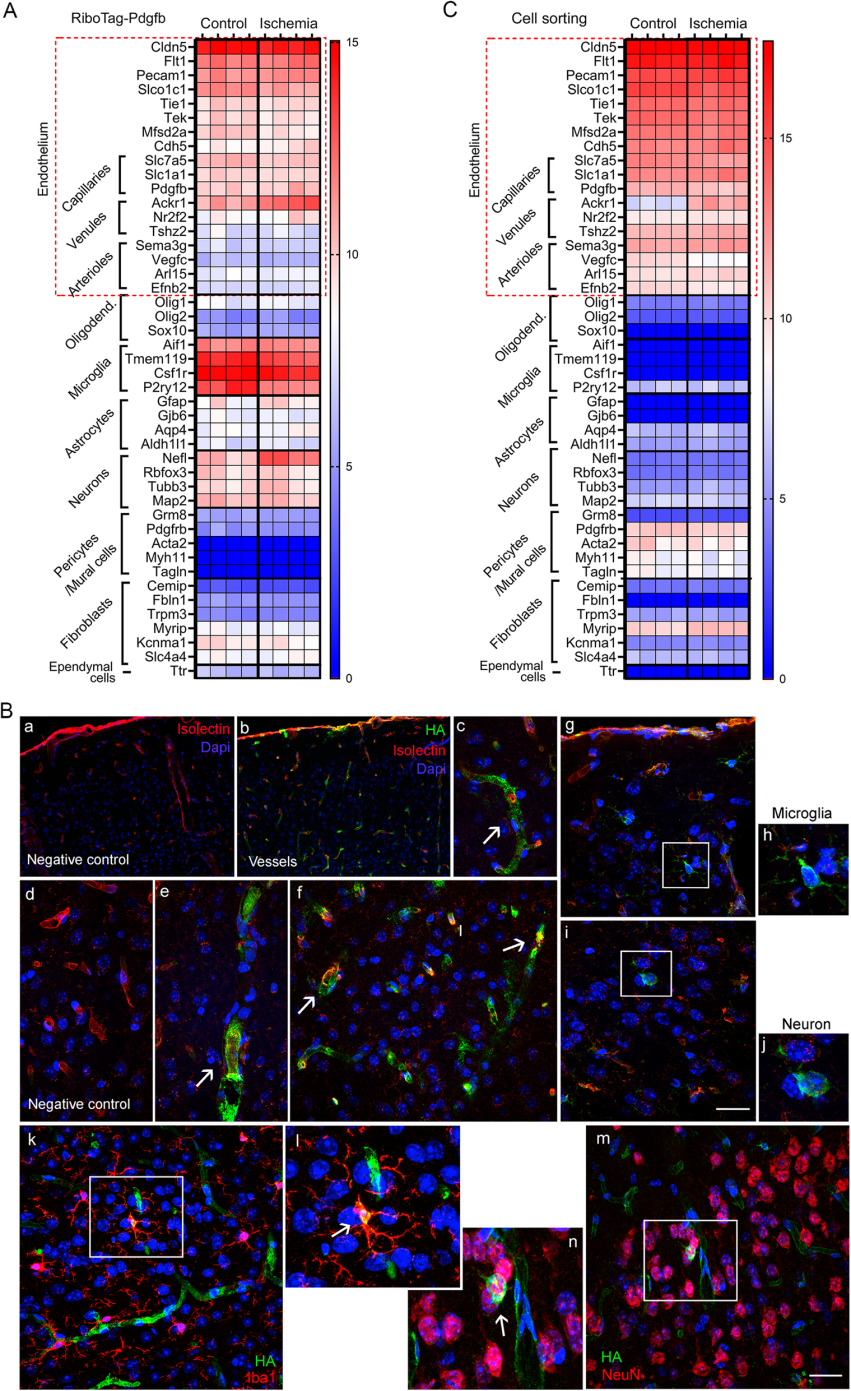
Expression cell types markers in the mRNA obtained from *Pdgfb*^*icreER*^*:Rpl22*^*HA*^ mice and isolated CD31^+^ cells. **A**, **C** Gene expression data obtained from RNAseq data shown in Figs. 1 and 2 corresponding to *Pdgfb*^*icreER*^*:Rpl22*^*HA*^ mice (**A**) and sorted CD31^+^ cells (**C**), respectively. Both mRNAs are enriched in endothelial cell markers, mainly capillaries and venules. mRNA from *Pdgfb*^*icreER*^*:Rpl22*^*HA*^ mice contained gene markers of microglia and neurons and, to a lower extent, fibroblasts. The mRNA from CD31^+^ cells contained mRNA markers of pericytes and/or mural cells. Each lane corresponds to the value from a different control or ischemic sample. **B** Immunofluorescence of cortex of *Pdgfb*^*icreER*^*:Rpl22*^*HA*^ mice stained with isolectin (red) to visualize brain vessels and anti-HA antibody (green). Nuclei are stained with DAPI (blue). The HA antibody mainly labels blood vessels (b), which are not detected in the immunofluorescence control with no anti-HA antibody (a). (c–f) show vessels at higher magnification, and the negative control is shown in (d). HA^+^ cells with morphology of microglia (g, h) or neurons (i, j) were occasionally detected. Co-localization was verified by double immunofluorescence of HA (green) with a microglial marker (Iba-1, red in k, l) or a neuronal marker (NeuN, red in m, n). Panel **l** and n are magnifications of the squares marked in panels *k* and* m*, respectively. Scale bar: ‘a, b’ = 80 μm; ‘c–g, i, k, m’ = 25 μm; ‘h, j, l, n’ = 12 μm

We next checked the enrichment of endothelial cell markers in our data sets by examining the expression of a selection of genes typical of different zones of the vascular tree, including general endothelial markers and markers associated with arterioles, venules, and capillaries [5]. In addition, we included markers of diverse brain cell types to assess putative contamination. The mRNA obtained from *Pdgfb*^*icreER*^*:Rpl22*^*HA*^ mice was enriched in capillaries and venules compared with arterioles (Fig. 3A), as expected given the higher expression of Pdgfb in the former vessels. However, we also detected markers of other cell types, notably microglia and neurons (Fig. 3A). This effect may be attributable to the fact that some Pdgfb expression was reported in neurons [20] and microglia [21]. To validate this possibility, we carried out immunofluorescence with anti-HA antibodies to visualize the cells tagged with HA in the *Pdgfb*^*icreER*^*:Rpl22*^*HA*^ mice after tamoxifen administration. HA^+^ staining was mainly observed in blood vessels (Fig. 3Ba–f). However, a few HA^+^ cells with morphological features of microglial cells (Fig. 3Bg, h) or neurons (Fig. 3Bi, j) were occasionally detected. These results were confirmed with double-immunofluorescence with HA and markers of either microglia (Iba-1) (Fig. 3Bk, l) or neurons (NeuN) (Fig. 3Bm, n).

Regarding the RNAseq data set obtained after CD31^+^ cell sorting, we found enrichment in gene markers of the different types of vessels, including capillaries, venules, and arterioles, while there was no expression of the studied mRNA markers of other cells except for low expression of pericyte markers (Fig. 3C), suggesting some contamination of CD31^+^ cells with pericytes during cell sorting. We validated the expression of a few of the above genes by RT-PCR in mRNA obtained either from sorted CD31^+^ endothelial cells, the RiboTag technique, or whole cortical tissue of independent groups of mice. Both endothelial-specific mRNA techniques showed enrichment of *Pecam1* compared to total tissue. Still, again, we detected *Pdgfrb* in both preparations, and the neuronal marker *Tubb3* in mRNA of the RiboTag technique (Additional file 5: Fig. S3). Altogether, these findings suggested that the mRNA isolated by CD31^+^ cell sorting was highly enriched in endothelial cells from different zones of the vascular tree but may contain some contamination of mRNA from pericytes, whereas the mRNA obtained from the *Pdgfb*^*icreER*^*:Rpl22*^*HA*^ mice contained transcripts from neurons and microglia.

### Identification of a translatomic module in brain endothelial cells responsive to acute ischemia

**Fig. 4:**
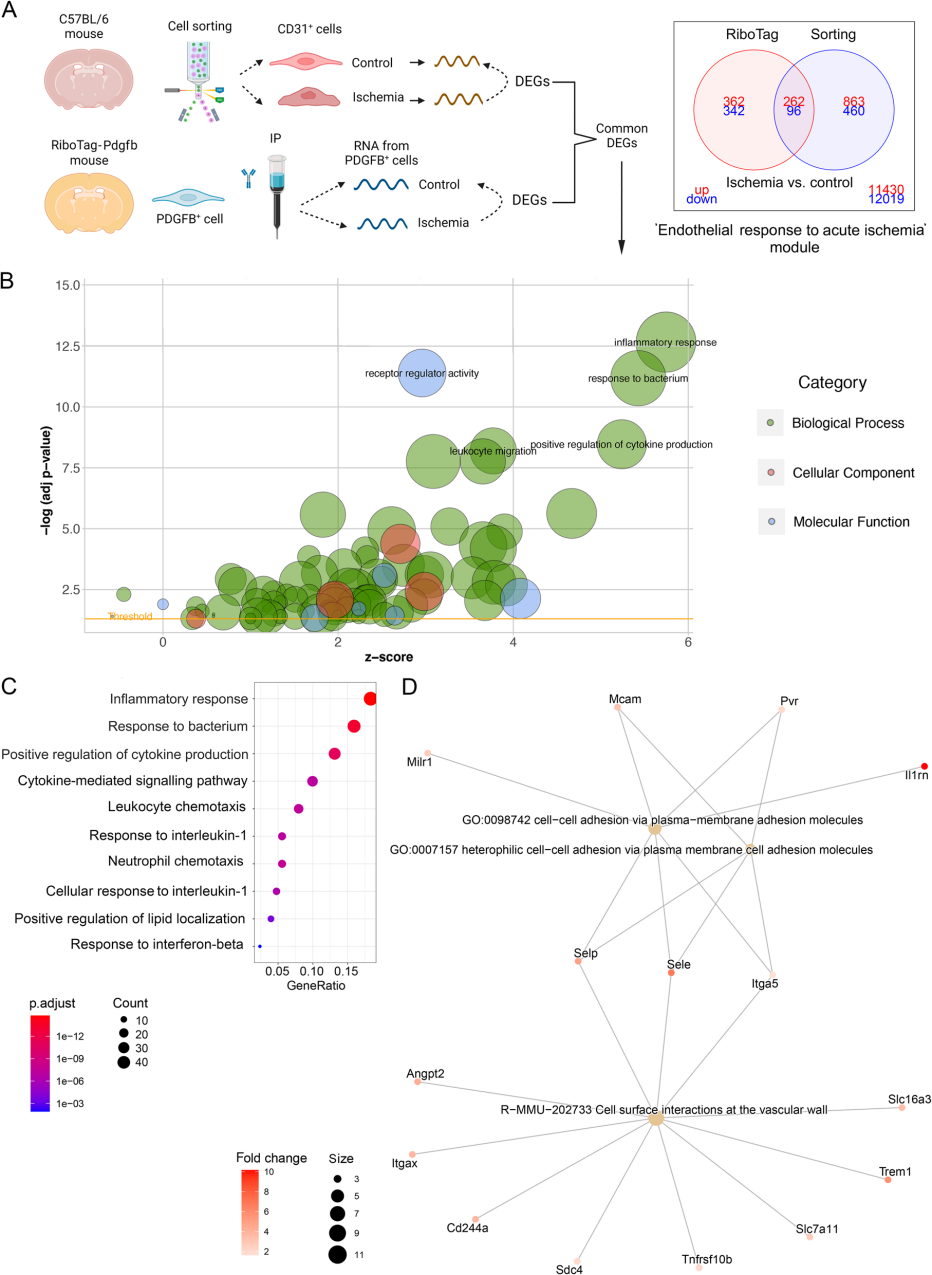
Analysis of ischemia-induced DEGs common to RNAseq data sets of *Pdgfb*^*icreER*^*:Rpl22*^*HA*^ mice and sorted CD31^+^ cells. **A** Brain endothelial cell mRNA was obtained from either the *Pdgfb*^*icreER*^*:Rpl22*^*HA*^ mice (RiboTag) or sorting CD31^+^ cells (Sorting) 24h post-ischemia or controls (image created with BioRender.com). DEGs induced by ischemia in each technique were identified, and the DEGs that were common to both techniques were determined. We detected 262 genes upregulated, whereas 96 genes were downregulated in both techniques (Additional file 6: Table S3). We termed this group of genes the ‘*Endothelial response to acute ischemia’* module. **B** Representative Reactome pathways enriched after ischemia in this module. Color code indicates fold change of ischemic vs. control and the size of the core dots indicate the number of DEGs in the network. **C** Top GO pathways enriched after ischemia in this module (Additional file 7: Table S4). **D** Illustrates genes and pathways upregulated after ischemia in the cell membrane, including ischemia-responsive genes previously identified in the endothelium, such as P- and E-selectins (*Selp, Sele)*, and new genes, such as *Mcam* and *Pvr*, amongst others

Comparison of both techniques showed a higher number of DEGs using the cell sorting approach than the RiboTag approach, as expected, since the latter technique only selects mRNA associated with ribosomes (Fig. 4A). As a strategy to identify the most robust translatomic changes induced by ischemia in endothelial cells, we decided to take a conservative approach to increase the chances of identifying genes that may have functional effects in endothelial cells in response to ischemia minimizing contamination with transcripts from other cell types. Therefore, we focused on the common genes that were differentially expressed after ischemia vs. control in both studies (Fig. 4A). In this way we concentrated in genes that were part of the translatome of endothelial cells, and enriched the data with DEGs from endothelial cells, mainly derived from capillaries and venules. In this procedure, there is a possible loss of some genes with weak or more variable differential expression in at least one of the two methodological strategies. Assuming this limitation, we identified a set of 358 common DEGs in the endothelial cells, comprising 262 genes upregulated and 96 genes downregulated by ischemia (Fig. 4A, Additional file 6: Table S3) that we globally termed ‘*Endothelial response to acute ischemia’* module. Gene Ontology (GO) analysis of this group of DEGs showed enrichment of pathways mainly related to inflammatory and innate immune responses, as illustrated by the most significant biological process (Fig. 4B) and the 10 most significant pathways (Fig. 4C, see full list in Additional file 7: Table S4). Downregulated genes included *Alpl*, a tissue-nonspecific alkaline phosphatase, and carbonic anhydrases *Car4* and *Car5a* with important metabolic regulatory functions.

The inflammatory reaction was highlighted by the response to interleukin-1 and leukocyte attraction and adhesion pathways, with upregulation of genes encoding cytokines, chemokines and adhesion molecules (Fig. 4B, C). The most upregulated chemokines and their receptors were Ccl2, Ccl3, Ccl7, Ccl9, Cxcl3, Cxcl10, and Cxcl16, and the atypical chemokine receptors Ackr1 and Gpr182. Gene set enrichment analysis with TopGO enabled visualization of functional profiles for gene and gene clusters highlighting hierarchical relations among inflammatory pathways. The most relevant inter-related pathways corresponded to cytokine and chemokine activity and receptor binding; particularly CCR2 and immunoglobulin Fc-gamma receptor I complex binding (Additional file 8: Fig. S4). An important enriched pathway was the interleukin-6 (IL-6) family signaling pathway, which induces a gene expression cascade for several chemokines, cytokines, and other inflammatory mediators following activation of the transcription factor Stat3 that was upregulated in the ischemic endothelium. Previous work demonstrated the involvement of Stat3 in long-term functional recovery after brain ischemia by inducing angiogenesis, extracellular matrix remodeling, and neuroplasticity [22]. In our study, the gene coding for a negative regulator of this pathway, *Socs3*, was also highly overexpressed in endothelial cells after ischemia (Additional file 6: Table S3). Moreover, we detected endothelial upregulation of Strawberry notch homolog 2 (*Sbno2*), which is a negative regulator of IL-6 signaling [23], suggesting a controlled regulation of the active signaling pathways. Ischemia also induced strong upregulation of the gp130 cytokine family, interleukin-11 (*Il11*) (Additional file 6: Table S3), which promotes proliferation of cells expressing the gp130/IL11RA receptor and it is involved in cardiovascular fibrosis [24]. Other upregulated pathways (Additional file 7: Table S4) included the ‘*Regulation of lipid localization*’ as well as the ‘*Response to interferon (IFN)-beta’* (GO:0035456) (Fig. 4C), angiogenesis (e.g., GO:0045765), extracellular matrix organization (e.g., GO:0030198), and oxidative stress (e.g., GO:1900407).

### Upregulation of genes encoding endothelial cell surface molecules

Molecules upregulated at the ischemic endothelial surface could be targets for pharmacological intervention either for blockade or to guide bioparticles to the ischemic vessels through functionalizing antibodies. Reactome pathway analysis highlighted the ischemia-induced enrichment in ‘*Cell surface interactions at the vascular wall’* (Fig. 4D). Upregulated genes in this pathway included genes encoding molecules typical of the endothelial surface that have already been described after ischemia, such as angiopoietin 2 (*Angpt,* Ang2), and E-selectin (*Sele*) and P-selectin (*Selp*) adhesion molecules (Fig. 4D). Some of these genes were also upregulated in the ‘*Heterophilic cell–cell adhesion *via* plasma membrane cell adhesion molecules’* pathway (GO:0007157) and the ‘*Cell–cell adhesion *via* plasma-membrane adhesion molecules*’ (GO:0098742) (Fig. 4D). In addition, within these pathways we detected the upregulation of genes encoding cell surface receptors with immunomodulatory properties that are less known in the context of cerebral ischemia. The latter genes included tumor necrosis factor receptor superfamily member 10b, *Tnfrsf10b*; the pattern recognition receptor triggering receptor expressed on myeloid cells *Trem1*, the poliovirus receptor *Pvr,* and melanoma cell adhesion molecules *Mcam* (Fig. 4D; Additional files 6 and 7: Tables S3 and S4). The immunoregulatory functions of the molecules encoded by these genes and their surface expression make them novel putative drug targets.

**Fig. 5:**
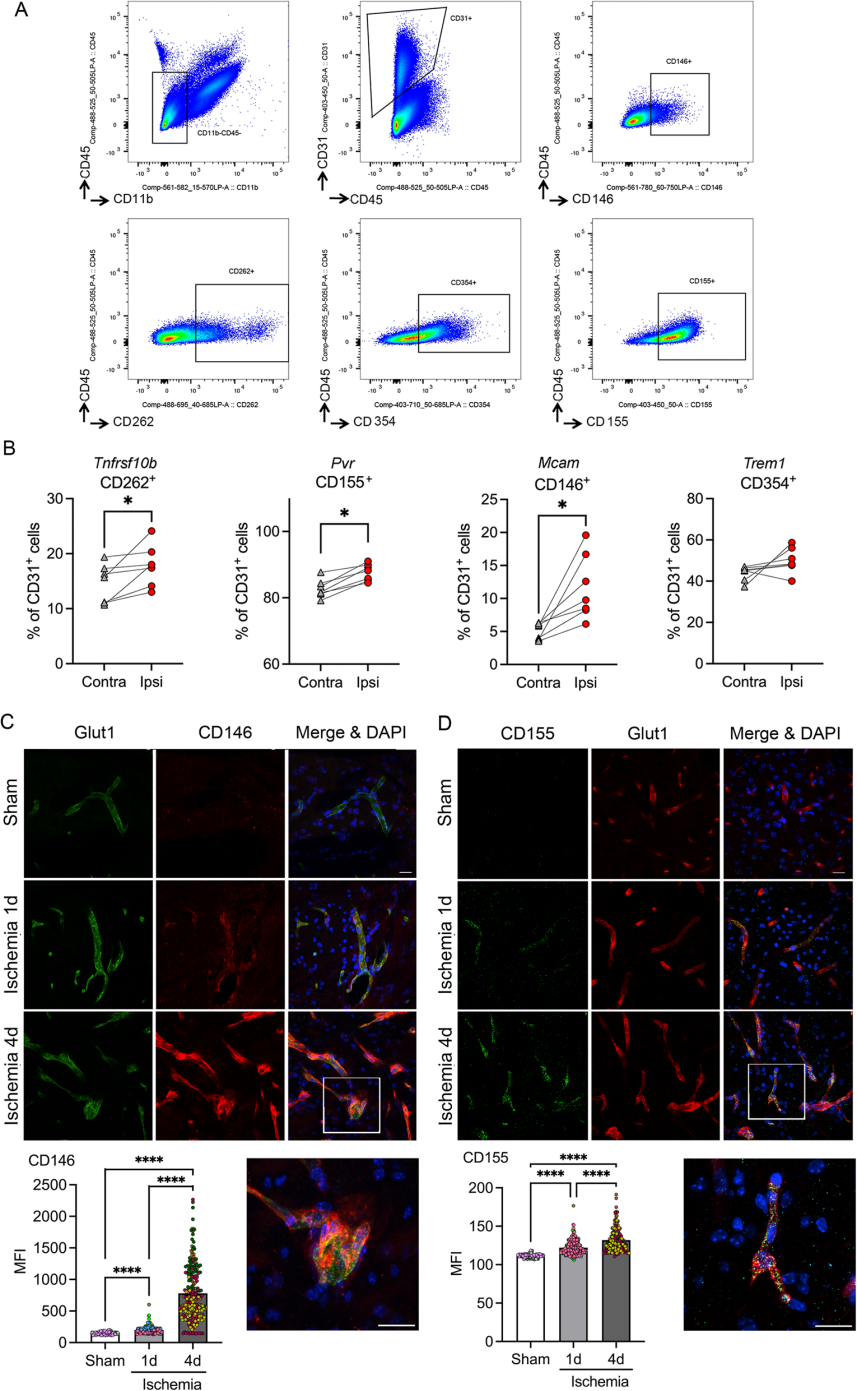
Ischemia-induced upregulation of the expression of certain immunomodulatory membrane receptors as assessed by flow cytometry. **A** Gating strategy for flow cytometry analysis. **B** Quantification of the % of CD31^+^ endothelial cells expressing the receptors in the contralateral (contra) and ipsilateral (ipsi, ischemic) hemispheres 24h post-ischemia (*n* = 6 per group) by flow cytometry. Ischemia increased the % of CD31^+^ cells expressing CD262 (*Tnfrsf10b)* (*p = 0.0156), CD155 (*Pvr*) (*p = 0.0156), and CD146 (*Mcam*) (*p = 0.0156). Changes in the % of CD31^+^ cells expressing CD354 (*Trem1*) (*p* = 0.1562) were not significant. Analysis was performed with the Wilcoxon matched-pairs signed rank test. **C**, **D** Immunofluorescence for CD146 (red in **C**) and CD155 (green in **D**) in the brain vessels (Glut1^+^) of sham-operated and ischemic mice at 1- and 4-day post-ischemia. Nuclei are stained with DAPI (blue). Scale bar: 20 μm. Images in the bottom row are magnifications of the squares shown in the merged images at day 4 post-ischemia. Quantification of mean fluorescence intensity (MFI) of CD146 and CD155 in brain vessels (number of vessels ranging from 145 to 250 obtained from 3 to 5 mice per group) ****p < 0.0001 (Kruskal–Wallis and Dunn’s tests)

We validated the endothelial protein expression and ischemia-induced upregulation of some of these molecules by flow cytometry (Fig. 5A). The proportion of CD31^+^ endothelial cells expressing CD262 (*Tnfrsf10b*), the poliovirus receptor CD155 (*Pvr*), and CD146 (*Mcam*) increased 1 day after ischemia (Fig. 5B), whereas the expression of CD354 (*Trem1*) only showed a non-significant trend to increase (Fig. 5B). Ischemia-induced Mcam (Fig. 5C) and CD155 (Fig. 5D) protein expression in brain endothelial cells was further demonstrated by immunofluorescence showing the increase in mean fluorescence intensity in vessels starting 1 post-ischemia and increasing further at 4 days (Fig. 5C, D).

### Inflammation and lipid accumulation in the endothelium

**Fig. 6:**
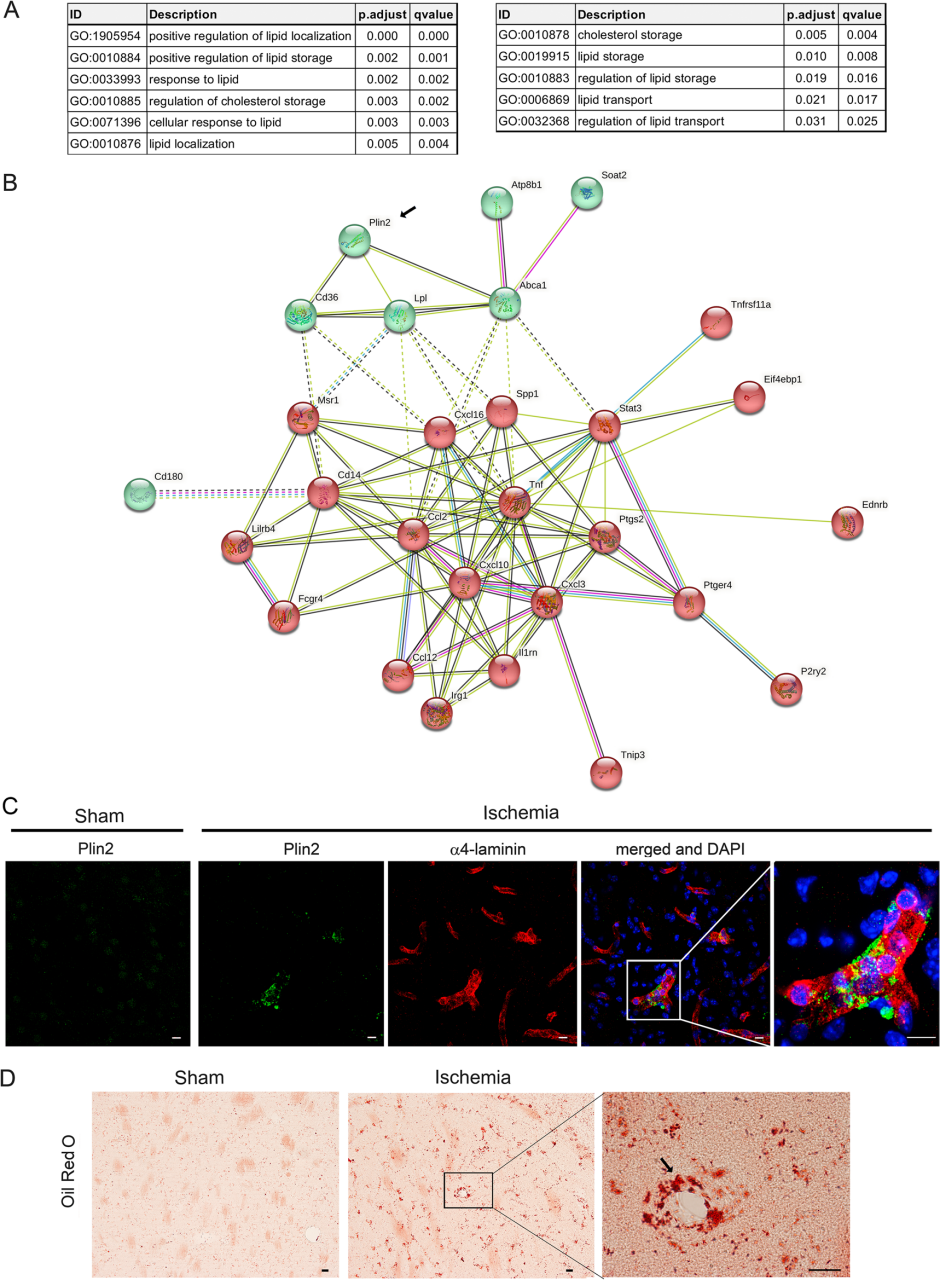
Ischemia-induced enrichment of pathways related to lipid storage. **A** List of representative pathways related to lipid transport and storage that are enriched after ischemia in the ‘Endothelial response to acute ischemia module’ (Fig. 4A). **B** Upregulated genes in the above pathways are illustrated by the STRING representation of protein networks. STRING analysis identified two strongly inter-related protein clusters. Plin2 (arrow) is a structural protein of lipid droplets. **C** Immunostaining of Plin2 in sham-operated mice (*n* = 3) and ischemic mice (1-day post-ischemia) (*n* = 4) shows the presence of Plin2 in some brain vessels of the ischemic hemisphere. **D** Oil Red O staining illustrates the presence of lipid droplets in brain vessels of the brain tissue 1 day after ischemia but not sham-operation. Images on the right side in **C** and **D**, are magnifications of the zone marked with squares in the previous images. Scale bar: 10 μm (**C**) and 20 μm (**D**)

Accompanying inflammation and innate immune responses, enrichment of pathways involving lipid regulation and storage were also noticeable in the endothelial cells after ischemia, as illustrated above by the ‘*Positive regulation of lipid localization’* pathway (Fig. 4C). Additional lipid-related pathways were significantly enriched after ischemia (Fig. 6A, Additional file 7: Table S4). Clustering analysis (String) of the genes in the above pathways identified two clusters (Fig. 6B), one related to lipid transport, storage, and regulation (Fig. 6B, green) and another cluster (Fig. 6B, red) associated with lipid-mediated inflammatory responses enriched in pathways, such as ‘*Response to lipopolysaccharide’* (GO:0032496). These results suggest that lipid metabolism is perturbed in endothelial cells after ischemia in association with the inflammatory response. Among the lipid regulatory genes, *Plin2* was strongly upregulated in the ischemic endothelium (Fig. 6B). This gene encodes Perilipin 2 or Adipose Differentiation-Related Protein (ADFP), which is a structural component of lipid droplets (LDs) [25]. LDs are cell fat storage organelles typical of adipocytes that can be generated by other cells under a variety of stimuli. We validated the ischemia-induced endothelial upregulation of Plin2 expression at the protein level by immunofluorescence in brain tissue section 1-day post-ischemia (Fig. 6C). Moreover, staining with Oil Red O showed the presence of lipid droplets in endothelial cells after brain ischemia (Fig. 6D).

## Discussion

This study reports a robust set of translatomic changes in endothelial cells of the cerebral microvasculature 24h after transient brain ischemia in mice. The study validates previous findings and identifies new DEGs in response to cerebral ischemia/reperfusion in the vascular endothelium, many of them bearing immunomodulatory functions. We employed two different strategies to obtain endothelial mRNA from the adult mouse brain, i.e., the *Pdgfb*^*icreER*^*:Rpl22*^*HA*^ mouse and the extraction of the RNA from CD31^+^ endothelial cells isolated by FACS. Enrichment analysis showed ischemia-induced modulation of similar pathways in both techniques. Moreover, our study confirmed, in both experimental approaches, the enrichment of genes described to be associated with BBB breakdown [6]. However, less DEGs were found with the RiboTag technique than with the cell sorting technique, since the translatome is more restrictive than the transcriptome. By examining the presence of typical gene markers of brain non-endothelial cell types, we detected some contamination of neuronal and microglial genes in the mRNA obtained from *Pdgfb*^*icreER*^*:Rpl22*^*HA*^ mice. Although Pdgfb is mainly expressed in endothelial cells, Pdgfb expression has been also reported in neurons [20] and microglia [21]. We validated these findings by immunofluorescence. In turn, we detected the presence of mRNA transcripts corresponding to pericytes in the samples obtained by FACS of CD31^+^ endothelial cells. The technical difficulties in obtaining very pure endothelial mRNA from the adult mouse brain devoid of any contamination with mRNA from other cell types were partly resolved by merging the data sets and selecting the common DEGs in both experimental techniques. This strategy increased the robustness of the selection of genes differentially expressed in endothelial cells after brain ischemia and focused on the translatome.

A recent transcriptomic study showed *Spp1* upregulation in the ischemic brain and involvement of the encoded protein Osteopontin (OPN) in BBB damage [7]. Accordingly, *Spp1* was among the genes of the described BBB dysfunction module [6], and *Spp1* was strongly upregulated in both of our methodological strategies. Moreover, we found downregulation of *Alpl*, encoding tissue nonspecific alkaline phosphatase, which dephosphorylates OPN reducing its activity [26]. Ischemia-induced endothelial dysfunction was illustrated by several other genes and pathways, for instance dysregulation of lipidic pathways and upregulation of *Angpt2*, which was included in the BBB dysfunction module [6]. A variety of matrix metalloproteinases (MMPs) and cathepsin peptidases (CTSs), challenge the integrity of the BBB after ischemia/reperfusion [27, 28]. We found high upregulation of *Mmp12* and *Mmp13*, and *Ctsb* in endothelial cells. Moreover, the ’Oxidative stress’ pathway may also promote endothelial dysfunction and BBB damage.

Endothelial upregulation of genes typically related to immune functions is illustrated by activation of IFN programs, and we detected upregulation of genes encoding cell membrane proteins not previously reported in the ischemic endothelium. We validated by flow cytometry and immunofluorescence the upregulation of several membrane proteins that could be accessible targets for pharmacological intervention from the blood. We found a prominent increase in endothelial CD155 (Pvr) expression after ischemia. CD155 has been detected in human umbilical vein endothelial cells in culture and vessels of the human placenta and skin under physiological conditions, where it is expressed at cell junctions and mediates monocyte transendothelial migration [29]. Ischemia also increased the expression of CD262 (Tnfrsf10b), a protein involved in apoptosis that also induces the release of procoagulant microparticles in endothelial cells [30]. Moreover, ischemia induced a strong endothelial upregulation of the cell adhesion molecule Mcam (CD146). Mcam is involved in cell cohesion at the intercellular junctions, but under inflammatory conditions, Mcam^+^ endothelial cells may facilitate leukocyte infiltration. Indeed, Mcam^+^ endothelial cells promote infiltration of pathogenic CD4^+^ lymphocytes in a murine model of experimental autoimmune encephalomyelitis and multiple sclerosis lesions [31].

This study also reveals intricate connecting pathways, where innate immune and inflammatory responses converge with alterations of lipid metabolism in the endothelial cells. Furthermore, the gene expression alterations in cell lipid trafficking and storage were strongly associated with inflammatory responses reminiscent of the response to lipopolysaccharide. At the protein level we demonstrated the ischemia-induced expression of the lipid droplet structural protein Plin2 in endothelial cells and lipid droplet biogenesis. Likewise, we recently reported Plin2 induction and LD biogenesis in microglia after cerebral ischemia [12]. These findings suggest the possibility that modulating lipid trafficking mechanisms may regulate the inflammatory responses and endothelial cell dysfunction after ischemic stroke.

We studied the gene expression profile of endothelial cells in the acute phase of stroke, while the overall gene expression pattern following a stroke differs during the acute, subacute, and long-term post-injury phases [32]. Considering the evolving conditions after stroke, there is a definite need for additional research to examine endothelial responses at extended time intervals, which would facilitate investigations into angiogenesis and tissue repair processes. Moreover, further analyses of endothelial cells from stroke patients are guaranteed for human validation. Notably, recent studies profiling at the single cell level the major brain vascular and perivascular cells from the human brain provided valuable insight regarding specific human features. For instance, a study of the hippocampus of Alzheimer’s disease patients and controls identified in the vasculature risk genes thought to be microglia-specific in mice [33]. Another study of brain tissue from patients with arteriovenous malformations revealed an expanded diversity of perivascular cells in human but not mouse brain [34].

## Conclusion

Altogether, this study unveils robust translatome changes in the cerebral microvascular endothelium in the acute phase following an episode of brain ischemia highlighting the upregulation of genes related to inflammatory and immune responses, endothelial dysfunction, and lipid regulation, while weakening barrier function and integrity. These results also show upregulation of membrane components and lipid trafficking and storage molecules that were not previously described in the ischemic brain vessels. Thus, the study identifies new putative targets for therapeutic intervention that deserve further investigation. Collectively, these results can pave the way to the discovery of novel druggable targets to improve endothelial barrier function and regulate the inflammatory and immune responses of the brain vasculature to ischemic stroke.

### Supplementary Information

**Additional file 1: Figure S1.** Infarct volume and heatmaps of global endothelial DEGs. A) Infarct volume of the Pdgfb^icreER^:Rpl22^HA^ mice. B) Heatmap of DEGs between ischemic and control endothelial cell RNA obtained from the Pdgfb^icreER^:Rpl22^HA^ mice. C) Infarct volume of the group of mice used for cell sorting. Differences in infarct volume between groups shown in (A) and (C) were not statistically significant (Mann–Whitney test, p = 0.69). The graphs in (A) and (C) show values for individual mice and the median and interquartile range. D) Heatmap showing corresponding transcriptomic data from CD31^+^ cells obtained by cell sorting comparing ischemic vs. control tissue (n = 4 per group and technique).

**Additional file 2: Table S1.** Pathway enrichment analysis for the Pdgf-RiboTag technique

**Additional file 3: Table S2.** Pathway enrichment analysis for the CD31^+^ cell sorting technique.

**Additional file 4: Figure S2.** Comparison of DEGs in our data sets with published data focusing on BBB dysfunction. A) Endothelial specific and brain tissue specific genes reported by Cleuren et al. [19] were found in our data sets, but they were enriched in the cell sorting data vs. the Ribotag data. B) BBB breakdown is illustrated in brain tissue sections by IgG extravasation at 1-day and 4-day post-ischemia. Quantification of integrated optical density of the IgG signal showed increases in the ipsilateral vs. the contralateral hemisphere at day 1 (n = 6, *p = 0.019) and day 4 (n = 4, *p = 0.020) post-ischemia (Kruskal–Wallis test with Dunn’s multiple comparisons test). The graph shows a violin plot, where each value is represented together with the median and interquartile ranges. Ipsi: ipsilateral, Contra: contralateral. C) Comparison of ischemia induced DEGs with the BBB dysfunction module of genes reported by Munji et al. [6] using GSEA analysis. We identified 44 gene coincidences in the Ribotag data (C) and 74 genes in the CD31^+^ cell sorting data (E). Corresponding GSEA plot signatures for RiboTag data (D) and CD31^+^ cell sorting data (F) show good enrichment of the BBB dysfunction module in our data sets.

**Additional file 5: Figure S3. **Validation of expression of cell-type marker genes by RT-PCR. In independent groups of naïve mice (n = 3 per group) we obtained endothelial mRNA by the RiboTag technique using Pdgfb^icreER^:Rpl22^HA^ mice, or by CD31^+^ cell sorting, as before. We extracted mRNA and carried out RT-PCR for validation of expression of cell markers. We compared the results with mRNA extracted from whole brain tissue (cortex) of naïve mice (n = 4). Values are expressed as fold vs. total brain tissue. Results show that the two methods of endothelial RNA extraction are enriched in endothelial markers, such as CD31 (Pecam1) and Vegfc. However, the platelet derived growth factor receptor beta, Pdgfrb, a marker of pericytes, is also enriched, and Tubb3, a marker of neurons is enriched in the mRNA obtained from the Pdgfbi^creER^:Rpl22^HA^ mice, confirming some contamination with RNA from other cell types in each preparation. ***p < 0.001, **p < 0.01. Two-way ANOVA and Šídák's multiple comparisons test. Data are shown as the mean ± SD.

**Additional file 6: Table S3.** Gene sets differentially expressed in ischemic endothelium commonly found in the RiboTag technique and the CD31^+^ cell sorting technique.

**Additional file 7: Table S4.** Pathways commonly up- or down-regulated in the RiboTag technique and CD31^+^ cell sorting technique.

**Additional file 8: Figure S4.** Visualization of functional profiles for gene and gene clusters within DEGs of the ‘Endothelial response to acute ischemia’ module (related to Fig. 4) by means of gene set enrichment analysis with TopGO. Hierarchical relations among inflammatory/immune pathways are highlighted, since they predominated in the acute phase of stroke.

## Data Availability

The RNA-Seq data are accessible from the GEO repository of the National Center for Biotechnology Information, U.S. National Library of Medicine. The accession numbers for these data are GEO: GSE223714 for RNAseq of ischemic and control brain tissue of *Pdgfb*^*icreER*^*:Rpl22*^*HA*^ mice, and GEO: GSE223744 for RNAseq of sorted CD31^+^ endothelial cells obtained by FACS from brain of ischemic and control mice. Other data sets will be available from the corresponding author upon reasonable request.

## Abbreviations

Angpt: Angiopoietin
BBB: Blood–brain barrier
Car: Carbonic anhydrases
CTS: Cathepsin peptidase
DAPI: 4′,6-Diamidino-2-phenylindole
DEG: Differentially expressed genes
DPBS: Dulbecco’s phosphate-buffered saline
FACS: Fluorescence-activated cell sorting
GEO: Gene Expression Omnibus
GO: Gene ontology
HA: Hemagglutinin
HBSS: Hank’s balanced salt solution
IL: Interleukin
IFN: Interferon
MCA: Middle cerebral artery
MCAM: Melanoma cell adhesion molecule (CD146)
MHC: Major histocompatibility complex
MMP: Matrix metalloproteinase
MRI: Magnetic resonance imaging
OPN: Osteopontin
PBS: Phosphate-buffered saline
Plin2: Perilipin2
PCA: Principal components analysis
Pdgfb: Platelet-derived growth factor-beta
Pvr: Poliovirus receptor (CD155)
R-MMU: Reactome pathway
Mus: Musculus
RNAseq: RNA sequencing
ROS: Reactive oxygen species
Rpl22: 60S Ribosomal protein L22
RRID: Research resource identifiers
VE: Vascular endothelial
VEGF: VE growth factor
