# Supplementary figures and images for: Transcriptomics and translatomics identify a robust inflammatory gene signature in brain endothelial cells after ischemic stroke
Source: J Neuroinflammation. 2023 Sep 11;20:207. doi: 10.1186/s12974-023-02888-6 (PMC10494365; doi:10.1186/s12974-023-02888-6)

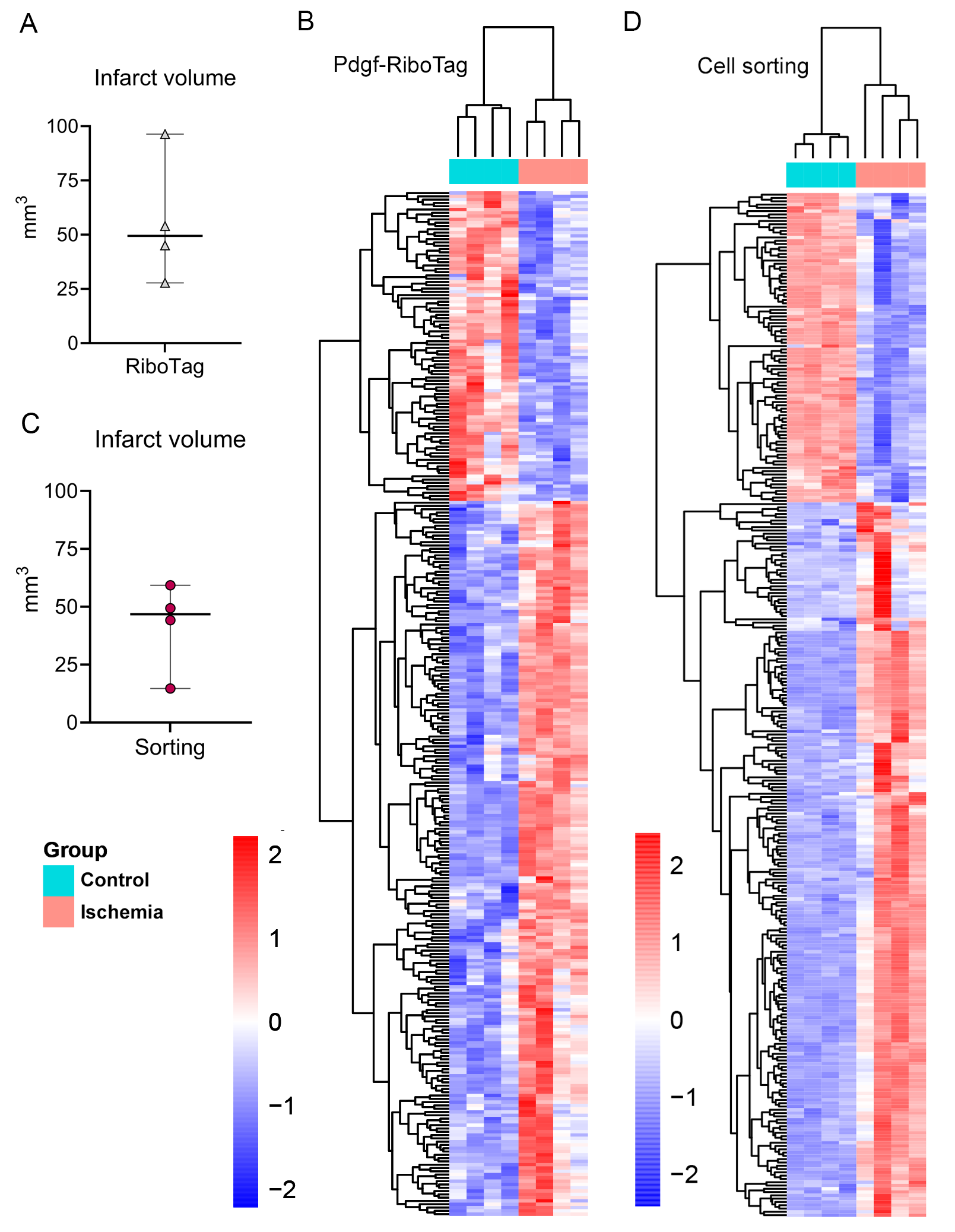

Supplement: Supplementary file 1 — Additional file 1: Figure S1. Infarct volume and heatmaps of global endothelial DEGs. A) Infarct volume of the PdgfbicreER:Rpl22HA mice. B) Heatmap of DEGs between ischemic and control endothelial cell RNA obtained from the PdgfbicreER:Rpl22HA mice. C) Infarct volume of the group of mice used for cell sorting. Differences in infarct volume between groups shown in (A) and (C) were not statistically significant (Mann–Whitney test, p = 0.69). The graphs in (A) and (C) show values for individual mice and the median and interquartile range. D) Heatmap showing corresponding transcriptomic data from CD31+ cells obtained by cell sorting comparing ischemic vs. control tissue (n = 4 per group and technique). [file 12974_2023_2888_MOESM1_ESM.tif]

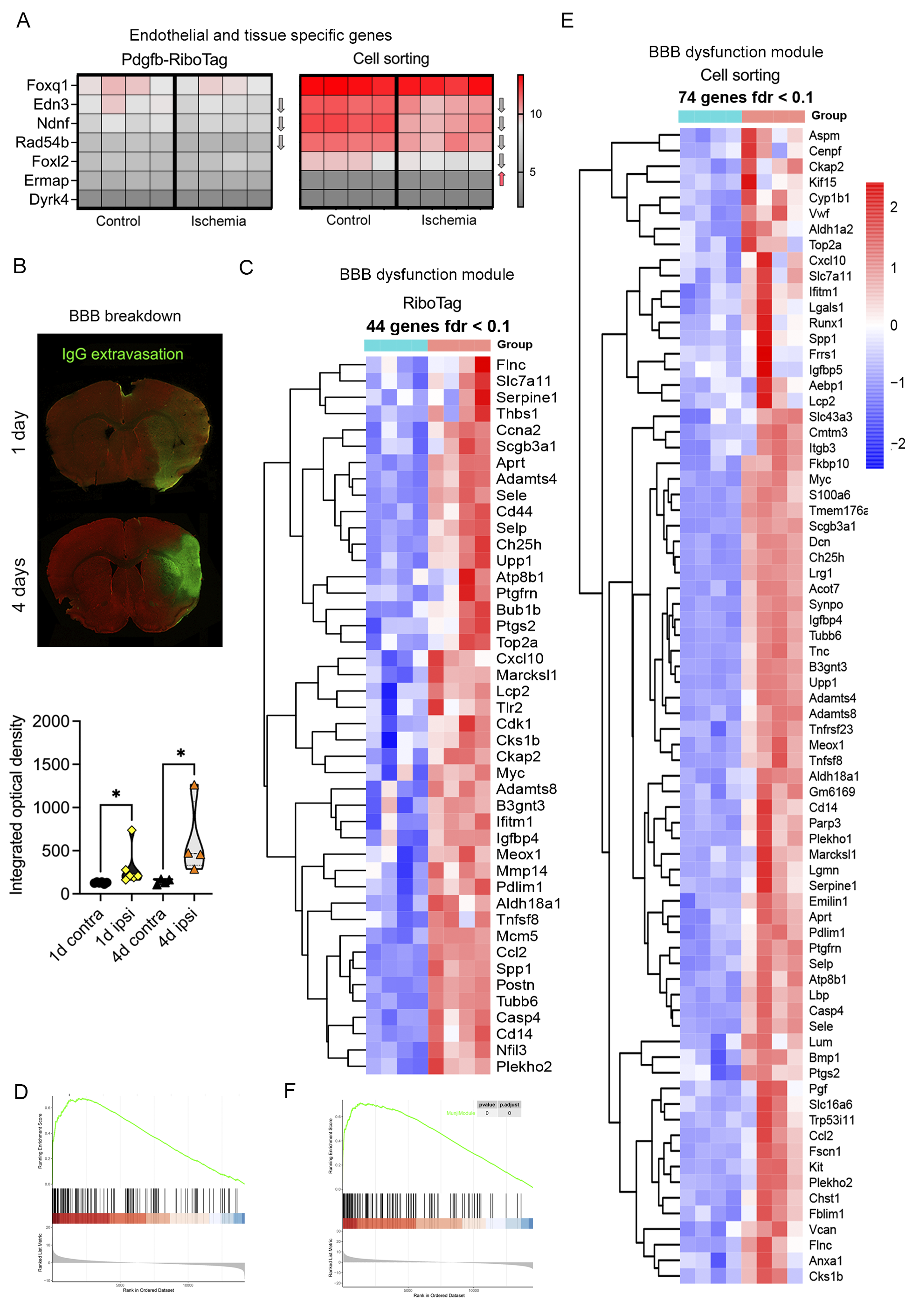

Supplement: Supplementary file 4 — Additional file 4: Figure S2. Comparison of DEGs in our data sets with published data focusing on BBB dysfunction. A) Endothelial specific and brain tissue specific genes reported by Cleuren et al. [19] were found in our data sets, but they were enriched in the cell sorting data vs. the Ribotag data. B) BBB breakdown is illustrated in brain tissue sections by IgG extravasation at 1-day and 4-day post-ischemia. Quantification of integrated optical density of the IgG signal showed increases in the ipsilateral vs. the contralateral hemisphere at day 1 (n = 6, *p = 0.019) and day 4 (n = 4, *p = 0.020) post-ischemia (Kruskal–Wallis test with Dunn’s multiple comparisons test). The graph shows a violin plot, where each value is represented together with the median and interquartile ranges. Ipsi: ipsilateral, Contra: contralateral. C) Comparison of ischemia induced DEGs with the BBB dysfunction module of genes reported by Munji et al. [6] using GSEA analysis. We identified 44 gene coincidences in the Ribotag data (C) and 74 genes in the CD31+ cell sorting data (E). Corresponding GSEA plot signatures for RiboTag data (D) and CD31+ cell sorting data (F) show good enrichment of the BBB dysfunction module in our data sets. [file 12974_2023_2888_MOESM4_ESM.tif]

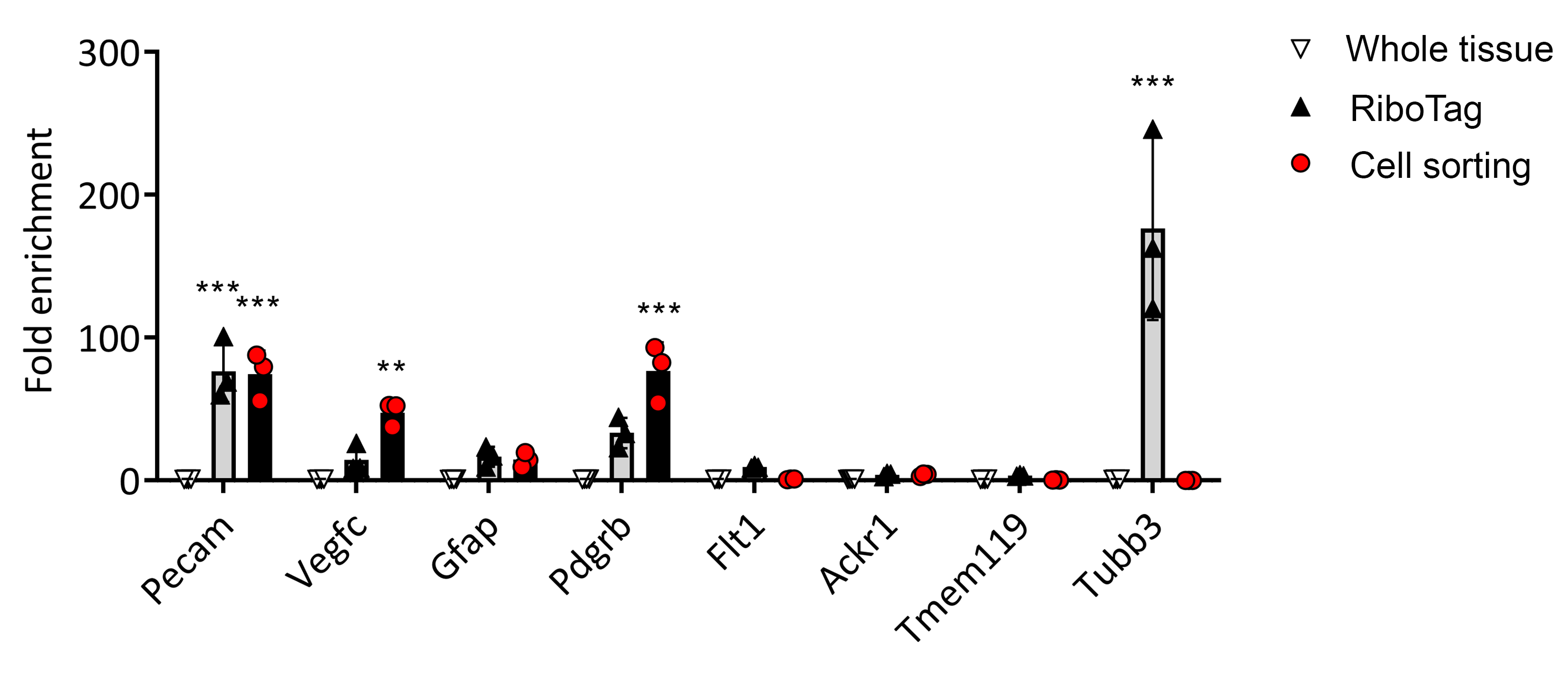

Supplement: Supplementary file 5 — Additional file 5: Figure S3. Validation of expression of cell-type marker genes by RT-PCR. In independent groups of naïve mice (n = 3 per group) we obtained endothelial mRNA by the RiboTag technique using PdgfbicreER:Rpl22HA mice, or by CD31+ cell sorting, as before. We extracted mRNA and carried out RT-PCR for validation of expression of cell markers. We compared the results with mRNA extracted from whole brain tissue (cortex) of naïve mice (n = 4). Values are expressed as fold vs. total brain tissue. Results show that the two methods of endothelial RNA extraction are enriched in endothelial markers, such as CD31 (Pecam1) and Vegfc. However, the platelet derived growth factor receptor beta, Pdgfrb, a marker of pericytes, is also enriched, and Tubb3, a marker of neurons is enriched in the mRNA obtained from the PdgfbicreER:Rpl22HA mice, confirming some contamination with RNA from other cell types in each preparation. ***p < 0.001, **p < 0.01. Two-way ANOVA and Šídák's multiple comparisons test. Data are shown as the mean ± SD. [file 12974_2023_2888_MOESM5_ESM.tif]

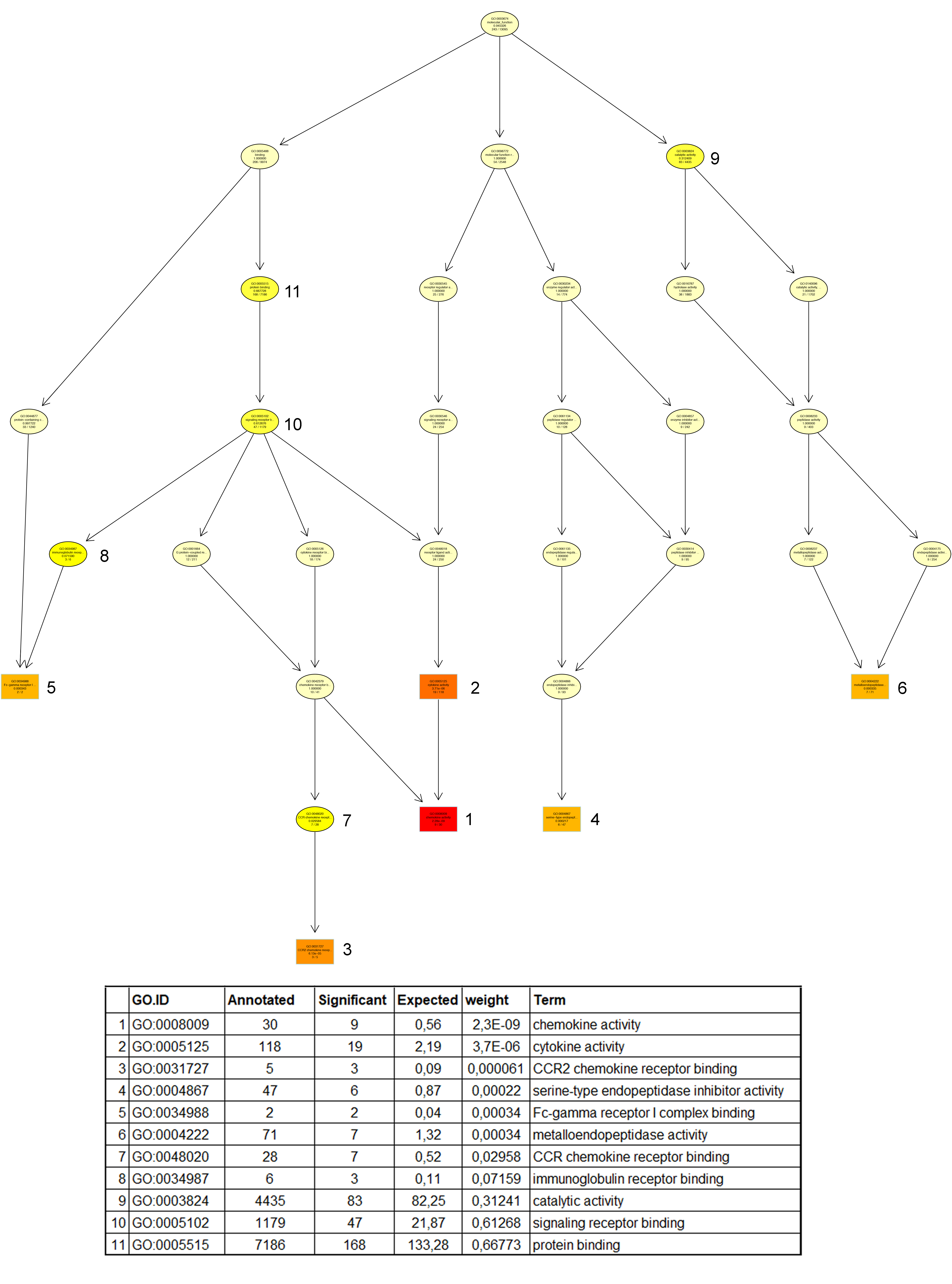

Supplement: Supplementary file 8 — Additional file 8: Figure S4. Visualization of functional profiles for gene and gene clusters within DEGs of the ‘Endothelial response to acute ischemia’ module (related to Fig. 4) by means of gene set enrichment analysis with TopGO. Hierarchical relations among inflammatory/immune pathways are highlighted, since they predominated in the acute phase of stroke. [file 12974_2023_2888_MOESM8_ESM.tif]
